# Intraoperative redosing of cefazolin and risk for surgical site infection in cardiac surgery.

**DOI:** 10.3201/eid0705.017509

**Published:** 2001

**Authors:** G Zanetti, R Giardina, R Platt

**Affiliations:** Brigham and Women's Hospital, Harvard Medical School, and Eastern Massachusetts CDC Prevention Epicenter, Boston, USA. Giorgio.Zanetti@chuv.hospvd.ch

## Abstract

Intraoperative redosing of prophylactic antibiotics is recommended for prolonged surgical procedures, although its efficacy has not been assessed. We retrospectively compared the risk of surgical site infections in 1,548 patients who underwent cardiac surgery lasting >240 min after preoperative administration of cefazolin prophylaxis. The overall risk of surgical site infection was similar among patients with (43 [9.4%] of 459) and without (101 [9.3%] of 1,089) intraoperative redosing (odds ratio [OR] 1.01, 95% confidence interval [CI] 0.70-1.47). However, redosing was beneficial in procedures lasting >400 min: infection occurred in 14 (7.7%) of 182 patients with redosing and in 32 (16.0%) of 200 patients without (adjusted OR 0.44, 95% CI 0.23-0.86). Intraoperative redosing of cefazolin was associated with a 16% reduction in the overall risk for surgical site infection after cardiac surgery, including procedures lasting <240 min.


### Intraoperative Redosing of Cefazolin and Risk for Surgical Site Infection in Cardiac Surgery

**Giorgio Zanetti,*† Richard Giardina,* and Richard Platt*‡**
*Brigham and Women's Hospital, Harvard Medical School, and the CDC Eastern Massachusetts Prevention Epicenter, Boston, Massachusetts, USA; †University Hospital, Lausanne, Switzerland; and ‡Harvard Medical School and Harvard Pilgrim Health Care, Boston, Massachusetts, USA

Intraoperative redosing of prophylactic antibiotics is recommended for prolonged surgical procedures, although its efficacy has not been assessed. We retrospectively compared the risk of surgical site infections in 1,548 patients who underwent cardiac surgery lasting >240 min after preoperative administration of cefazolin prophylaxis. The overall risk of surgical site infection was similar among patients with (43 [9.4%] of 459) and without (101 [9.3%] of 1,089) intraoperative redosing (odds ratio [OR] 1.01, 95% confidence interval [CI] 0.70-1.47). However, redosing was beneficial in procedures lasting >400 min: infection occurred in 14 (7.7%) of 182 patients with redosing and in 32 (16.0%) of 200 patients without (adjusted OR 0.44, 95% CI 0.23-0.86). Intraoperative redosing of cefazolin was associated with a 16% reduction in the overall risk for surgical site infection after cardiac surgery, including procedures lasting <240 min.

Surgical site infections are important causes of illness and resource utilization (1,2). Perioperative antibiotic prophylaxis is widely used to reduce their incidence. On the basis of pharmacokinetic considerations, most published guidelines recommend intraoperative redosing of the prophylactic antibiotic for procedures of prolonged duration to maintain effective antibiotic concentrations (1,3-6).

Support for intraoperative redosing of antibiotics has been inferred from observational studies in which increased duration of surgery was associated with increased risk for surgical site infection, as well as loss of the protective effect of prophylaxis over time (7-9). However, the actual clinical benefit of intraoperative antibiotic redosing has not been confirmed or quantified in either clinical trials or observational studies.

We therefore carried out a retrospective cohort study to assess the effect of intraoperative redosing of prophylaxis on the occurrence of surgical site infection after prolonged cardiac procedures. Cardiac surgery was chosen for the study because its duration is typically long enough to meet the threshold for redosing in most published guidelines and because it carries a substantial risk for surgical site infection (10,11).

### Methods

This retrospective cohort study involved patients operated on in the Division of Cardiac Surgery at Brigham and Women's Hospital, Boston, Massachusetts, from April 1, 1998, to September 30, 1999. The study population was restricted to patients who received a first preoperative 1-g dose of cefazolin beginning ≤90 minutes before incision of the skin and whose procedures lasted ≥240 minutes after that dose. This minimum duration was chosen because it was the redosing interval recommended by the hospital's guidelines during the study period. No antibiotic redosing was given for any procedures of shorter duration; they would therefore not have contributed to our study. Patients were excluded if they received therapeutic antibiotics at the time of surgery. Patients were included only once in the analysis.

Intraoperative redosing was defined as the administration of a second dose of cefazolin at any time before surgical closure. In addition to the preoperative dose and an intraoperative redose when applicable, patients typically had at least six additional 1-g doses of cefazolin prescribed during the postoperative period.

For each eligible operation, the following data were abstracted from the anesthesiologist's report: age and sex of patient, date and type of surgery, surgeon, and the time of all antibiotic administrations. In addition, data on reoperations during the same hospital stay (except if reoperation followed a diagnosis of a surgical site infection) were collected from the hospital information system. To avoid comparisons between small subgroups, surgeries were categorized as to whether they included coronary artery bypass grafting.

Surgical site infections were prospectively identified by modified National Nosocomial Infection Surveillance methods and criteria (1) by an infection control practitioner who did not know whether the patient had received an intraoperative redose of antibiotic. This method encompassed both inpatient components and postdischarge information from the surgeons' offices.

To compare the patients who had received intraoperative redosing of cefazolin with those who had not, we used the 2-sided Wilcoxon rank sum test for continuous variables and the chi-square test for proportions. The significance level was 0.05 in all tests. Significant univariate predictors

Address for correspondence: Giorgio Zanetti, Division of Infectious Diseases, University Hospital, 1011 Lausanne, Switzerland; fax: 41-21-314-1018; e-mail: Giorgio.Zanetti@chuv.hospvd.ch of surgical site infection were then candidates for inclusion in a logistic regression model that was built through a forward selection process (12). The absence or presence of intraoperative antibiotic redosing was always forced in the model, as was an interaction term of procedure duration and intraoperative redosing, as described below. The model was then tested for confounding by each of the excluded covariates. The Wald test was used to report the significance level of the predictors in the final model (13). The odds of surgical site infection were also compared for redosing, categorized as either absent, given after 240 min, or given within 240 min, with the likelihood ratio test used to assess deviance from linearity.

To investigate whether intraoperative redosing had different effects on the risk for infection across different procedure durations, we created an interaction term with duration (categorized as <300 min, 300 to 400 min, or >400 min) and intraoperative redosing. These thresholds were chosen before the analysis began. The likelihood ratio test was used to assess the significance of this interaction term. Statistical analyses were performed with SAS statistical software (SAS Inc., Cary, NC).

### Results

Among 2,751 cardiac operations performed from April 1998 through September 1999, 1,886 (69%) lasted >240 min from the time of preoperative administration of antibiotic prophylaxis. We excluded 214 procedures (11%) because the patients received antibiotics other than cefazolin for prophylaxis, 44 (2%) because cefazolin had been administered either after surgical incision or >90 min before; 17 (1%) because of ongoing antibiotic therapy; and 8 (0.4%) because the patients had already been included in the analysis. Data were available for 1,548 (97%) of the 1,603 eligible patients.

Intraoperative redosing of cefazolin was administered to 459 (30%) of the patients, including 276 (18%) who received it within 240 min. These patients were compared with those who did not receive redosing (Table 1). The mean duration of surgery, measured from the administration of the preoperative dose of antibiotic, was significantly longer (p = 0.0001) in patients who were redosed. The distribution of surgeons also differed between the two groups. Intraoperative redosing was not associated with any of the available covariates, i.e., age, sex, type of surgery, need for reoperation, or calendar date.

Surgical site infection was diagnosed in 144 (9.3%) patients. One third of these infections were deep. There was no statistically significant difference among surgeon-specific infection rates (range 7.7% to 11.3%). In the whole study population, the overall risk for infection was similar in patients who received intraoperative redosing of cefazolin (43 [9.4%] of 459) and in those who did not (101 [9.3%] of 1,089) (OR 1.01, 95% CI 0.70 - 1.47). Multivariate analysis showed that the risk for surgical site infection increased with patient age and procedure duration and was also higher in coronary artery bypass grafting surgery. The latter finding was expected because of the additional incision for vein graft harvesting. There was also a significant interaction between surgery duration and intraoperative redosing (p = 0.015); redosing was associated with a lower infection rate in the longer procedures but not shorter ones.

Table 1. Characteristics of patients undergoing cardiac surgery

| | Intraoperative antibiotic redosing | | |
|---|---|---|---|
| Variable | Yes (n = 459) | No (n = 1,089) | p |
| Mean age (range, years) | 65.2 (17–91) | 65.7 (20–92) | NS[a] |
| Mean duration of surgery[b] | 393 (241-900) | 345 (241–700) | 0.0001 |
| Male sex (%) | 328 (71.5) | 744 (68.3) | NS |
| Type of surgery | | | |
| CABG | 317 (69.1) | 784 (72.0) | NS |
| Others | 142 (30.9) | 305 (28.0) | |
| Reoperation[c] | 53 (11.6) | 105 (9.6) | NS |

[a]NS = not significant; CABG = coronary artery bypass graft.
[b]Minutes elapsed between administration of preoperative antibiotics and skin closure.
[c]Reoperation within the same hospital stay is included, except for surgery following a diagnosis of surgical site infection.

Independent predictors of surgical site infection were analyzed for two categories of procedure duration (Table 2). Intraoperative redosing had a significant protective effect only in procedures lasting >400 min, for which it was associated with a 0.44 odds ratio (OR; 95% confidence interval [CI] 0.23 to 0.86) of surgical site infection. This corresponds approximately to a 56% reduction in risk for infection among these procedures, resulting in a risk that does not differ significantly from that observed in surgery that lasted 240 to 400 min.

Table 2. Independent predictors of surgical site infections after cardiac surgery

| Predictor | Adjusted odds ratio for SSI[a] | 95% confidence interval | p[b] |
|---|---|---|---|
| Procedures lasting ≤400 min | | | |
| Age[c] | 1.2 | 1.00-1.45 | 0.049 |
| CABG surgery | 1.84 | 1.05-3.20 | 0.032 |
| Duration of surgery[d] | 1.38 | 1.00-1.82 | 0.032 |
| Intraoperative redosing of antibiotics | 1.27 | 0.80-2.02 | 0.319 |
| Procedures lasting >400 min | | | |
| CABG surgery | 2.2 | 1.05-4.61 | 0.036 |
| Intraoperative redosing of antibiotics | 0.44 | 0.23-0.86 | 0.016 |

[a]SSI: surgical site infection; CABG: coronary artery bypass graft.
[b]Wald test.
[c]Odds ratio for every additional decade of age.
[d]Odds ratio for every additional hour of surgery.

We also explored different redosing schedules during procedures of >400 min. There was a significant trend toward a lower risk for infection when redose was either not given, given after 240 min, or given within 240 min ($p = 0.001$).

Of the patients who received prophylaxis with cefazolin, 20% had a procedure that lasted >400 min. For all patients, including those with a procedure lasting <240 min, we estimate that the infection rate in the absence of any redosing would have been 9.4%. If every patient whose procedure lasted >240 min had been redosed, the expected infection rate would have been 7.9%, representing a 16% reduction in the overall risk of postoperative surgical site infection attributable to redosing. The distribution of this expected risk in operations more or less than 400 min in duration, with or without redosing, is shown in the Figure.

Since procedures had been arbitrarily categorized before inspection of the data according to a duration of more or less than 400 min, we analyzed the impact of intraoperative redosing with different thresholds of duration. The benefit of intraoperative redosing was significant for a boundary between 385 and 415 min. However, there was a general trend toward greater benefit for higher thresholds.

### Discussion

This retrospective study demonstrates that intraoperative redosing of cefazolin provided additional protection against surgical site infection among patients undergoing cardiac surgery lasting longer than approximately 6.5 to 7 h. Although this group includes only a minority of procedures, we estimate from our data that a strategy of redosing in all procedures >240 min long results in a 16% reduction in the overall infection rate in cardiac surgery. This rate in our study population was comparable with that reported by others (10,11,14,15). The benefit from redosing had been assumed but had not been proven, and the minimum duration at which redosing is beneficial had been derived from theoretical considerations. Redosing provided similar protection from both deep and superficial infections (data not shown).

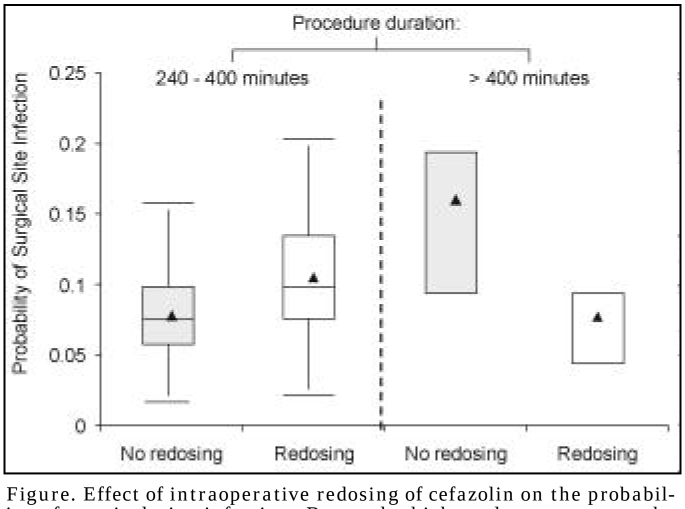

Figure. Effect of intraoperative redosing of cefazolin on the probability of surgical site infection. Box-and-whisker plots represent the probabilities of surgical site infection in 1,548 patients undergoing cardiac surgery, stratified by procedure duration, with or without intraoperative redosing of cefazolin. The probabilities for each member of the cohort were computed on the basis of redosing of antibiotic prophylaxis, the patient's age, and the type and duration of the procedure. The mean is represented by a triangle and the median by a bar within the boxes. There is no bar for procedures >400 min in duration because all the probabilities were clustered at the extremities of the boxes.

The positive association between duration of surgery and risk for surgical site infection has been reported (7-9). Our data show that this association persists even when antibiotic is redosed. This observation suggests that the risk related to duration not only reflects a diminution of antibiotic concentration over time but also may be a proxy for risk factors independent of antibiotic use, such as the technical difficulty of the procedure.

Guidelines usually recommend redosing intervals of 3 to 4 h for cefazolin (1,3-6). In a study on hysterectomy, for instance, a protective effect of prophylaxis was no longer observed when the operation lasted >3.3 h (8). The benefit extended beyond this threshold in our study, a finding that may reflect the markedly prolonged serum half-life of cefazolin during cardiopulmonary bypass. Although the half-life of cefazolin is 1.8 h in healthy persons (3), several studies have shown a slower elimination during cardiopulmonary bypass (14,16,17). Therefore, any benefit of redosing in noncardiac surgery may be observed for shorter procedures than in cardiac surgery.

This study has several limitations. Because of its retrospective design, the results were adjusted for a limited number of risk factors for surgical site infection, including surgery duration, age of the patient, and need for reoperation. Certain coexisting conditions, such as diabetes mellitus or obesity, smoking status, length of previous hospital stay, and a violation of asepsis during surgery, are among the predictors of surgical site infection that might confound our results, should they be related to the probability of an intraoperative antibiotic redose. Since the most plausible effect of a high-risk profile is to increase the likelihood of intraoperative redosing, adjustment for this profile would increase the apparent benefit. If patients undergoing more complicated (and therefore more infection-prone) procedures were less likely to be redosed for any reason, the effect of redosing would be overestimated. However, in that case, we would expect to see an effect for all procedures, not only longer ones. Our study also provides no information about the utility of additional doses of prophylaxis after surgery. Finally, our sample size limits the precision of our estimates, especially the ability to identify a precise threshold beyond which redosing is beneficial. Thus, we do not know whether similar protection could be obtained by redosing cefazolin only beyond the 400-min threshold. In an exploratory analysis of timing of the redose, there was a significant trend toward higher benefit when a redose was given within 240 min. Therefore, our results should not be used to support an extension of the 3- to 4-h redosing interval recommended by most guidelines (1,3-6).

We conclude that redosing of cefazolin prophylaxis for most cardiac procedures can prevent a substantial fraction of surgical site infections. It will be worthwhile to examine the effects of intraoperative redosing in other procedures.

This study was supported in part by cooperative agreement UR8/CCU115079 from the Centers for Disease Control and Prevention. Dr. Zanetti is supported by grants from the University Hospital of Lausanne and the Leenaards Foundation, Lausanne, Switzerland.

Dr. Zanetti is an associate hospital epidemiologist and infectious diseases attending physician at Lausanne University Hospital,

Lausanne, Switzerland. He is also a visiting scientist at Channing Laboratory, Brigham and Women's Hospital, Boston. His research focus includes optimization of antibiotic use, surgical site infection, and infection in cancer and intensive care unit patients.

### References

1. Mangram AJ, Horan TC, Pearson ML, Silver LC, Jarvis WR, the Hospital Infection Control Practices Advisory Committee. Guideline for prevention of surgical site infection, 1999. Infect Control Hosp Epidemiol 1999;20:250-78.
2. Kernodle DS, Kaiser AB. Postoperative infections and antimicrobial prophylaxis. In: Mandell GL, Bennett JE, Dolin R, editors. Principles and practice of infectious diseases. 4th ed. New York: Churchill Livingstone; 1995. p. 2742-61.
3. Dellinger EP, Gross PA, Barrett TL, Krause PJ, Martone WJ, McGowan JE, et al. Quality standard for antimicrobial prophylaxis in surgical procedures. Clin Infect Dis 1994;18:422-7.
4. Page CP, Bohnen JM, Fletcher JR, McManus AT, Solomkin JS, Wittmann DH. Antimicrobial prophylaxis for surgical wounds. Guidelines for clinical care. Arch Surg 1993;128:79-88.
5. ASHP Commission on Therapeutics. ASHP therapeutic guidelines on antimicrobial prophylaxis in surgery. Clin Pharm 1992;11:483-513.
6. Martin C, the French Study Group on Antimicrobial Prophylaxis in Surgery, the French Society of Anesthesia and Intensive Care. Antimicrobial prophylaxis in surgery: general concepts and clinical guidelines. Infect Control Hosp Epidemiol 1994;15:463-71.
7. Kaiser AB, Herrington JL, Jacobs JK, Mulherin JL, Roach AC, Sawyers JL. Cefoxitin versus erythromycin, neomycin, and cefazolin in colorectal operations. Importance of the duration of the surgical procedure. Ann Surg 1983;198:525-30.
8. Shapiro M, Munoz A, Tager IB, Schoenbaum SC, Polk BF. Risk factors for infection at the operative site after abdominal or vaginal hysterectomy. N Engl J Med 1982;307:1661-6.
9. Coppa GP, Eng K. Factors involved in antibiotic selection in elective colon and rectal surgery. Surgery 1988;104:853-8.
10. L'Ecuyer PB, Murphy D, Little JR, Fraser VJ. The epidemiology of chest and leg wound infections following cardiothoracic surgery. Clin Infect Dis 1996;22:424-9.
11. Sands K, Yokoe D, Hooper D, Tully J, Platt R. A multi-institutional comparison of surgical site infection surveillance by screening of administrative and pharmacy data. Proceedings of the 8th Annual Meeting of the Society for Healthcare Epidemiology of America; San Francisco, CA, 1999. Thorofare (NJ): Slack Inc.; 2000. Abstract M35.
12. Hosmer DWJ, Lemeshow S. Model building strategies and methods for logistic regression. In: Applied logistic regression. 2nd ed. New York: Wiley; 1989. p. 82-134.
13. Hosmer DWJ, Lemeshow S. Introduction to the logistic regression model. Testing for the significance of the coefficients. In: Applied logistic regression. 2nd ed. New York: Wiley; 1989. p. 11-8.
14. Maki DG, Bohn MJ, Stolz SM, Kroncke GM, Acher CW, Myerowitz PD. Comparative study of cefazolin, cefamandole, and vancomycin for surgical prophylaxis in cardiac and vascular operations. J Thorac Cardiovasc Surg 1992;104:1423-34.
15. Harbarth S, Samore MH, Lichtenberg D, Carmeli Y. Prolonged antibiotic prophylaxis after cardiovascular surgery and its effect on surgical site infections and antimicrobial resistance. Circulation 2000;101:2916-21.
16. Platt R, Munoz A, Stella J, VanDevanter S, Koster JK. Antibiotic prophylaxis for cardiovascular surgery. Ann Intern Med 1984;101:770-4.
17. Goldmann DA, Hopkins CC, Karchmer AW, Abel RN, McEnany T, Akins C, et al. Cephalotin prophylaxis in cardiac valve surgery. J Thorac Cardiovasc Surg 1977;73:470-9.
